# Retrospective study on MRI accuracy in assessing liver fibrosis among patients with chronic liver disease

**DOI:** 10.6026/973206300211643

**Published:** 2025-06-30

**Authors:** Subha Santhoshi Vanka, Nikita Rao Nalla, Shaheed Shaik, Navneeth Jayaprakash, Mohammed Zakiullah Shareef, Sneha Mahalpure

**Affiliations:** 1 Departments of Medicine, Madurai Medical College, Madurai, Tamilnadu 625020, India; 2 Department of Gastroenterology, Mahatma Gandhi Medical College and Research Institute, Puducherry, Tamilnadu, India; 3 Department of Radiology, Gandhi Medical College, Musheerabad, Secunderabad, Telangana, India; 4 Department of Internal Medicine, D.Y. Patil Medical College, Kolhapur, Maharashtra, India; 5 Department of Internal Medicine, AL Hada Armed Forces Hospital , AL Hada, TAIF, Kingdom of Saudi Arabia; 6 Department of Anatomy, NKP Salve Institute of Medical Sciences, Nagpur, Maharashtra, India

**Keywords:** Liver fibrosis, chronic liver disease, MRI elastography, non-invasive assessment, fibrosis staging, diagnostic accuracy

## Abstract

Magnetic resonance imaging (MRI) is increasingly being utilized for the non-invasive liver fibrosis evaluation among chronic liver disease (CLD) patients. Therefore, it is of interest to assess the staging and diagnostic accuracy of MRI for liver fibrosis compared to histopathological outcome in 150 patients with CLD. MRI data were analyzed with advanced techniques such as elastography and T1/T2 mapping and these results were matched against fibrosis stages ascertained through biopsy. The sensitivity and specificity of MRI in detecting extensive fibrosis ≥F2 were 88% and 91%, respectively. Fibrosis scores determined by MRI were correlated with histopathological stage with r = 0.87, p < 0.01. The findings revealed MRI as an effective non-invasive modality for liver fibrosis that, complementarily, decreases the position of invasive biopsy in patients with CLD.

## Background:

Chronic liver disease is a serious global health issue and most of the cases evolve to liver fibrosis, cirrhosis and their complications [1]. Liver fibrosis staging is crucial for staging, prognosis and therapeutic treatment. Liver biopsy has been used as the reference for all other tests for fibrosis diagnosis and staging [2]. But it is invasive with a potential for associated complications and sampling variability is a built-in drawback. Thus, safe non-invasive diagnostic options urgently need to become available. MRI has proven to be one of the more promising modalities to evaluate liver fibrosis, based on high resolution and sophisticated techniques such as MR Elastography, T1/T2 mapping and diffusion-weighted imaging [3]. These quantify the stiffness and structural alterations in the liver tissue and offer profound assessment of the extent of fibrosis, without invasive examination [4]. Numerous studies have established correlations with MRI images and histopathologic stages of fibrosis, rendering it a trustworthy substitute for fibrosis staging [5]. In the current research, the diagnostic usefulness of MRI in the diagnosis and staging of liver fibrosis in patients with CLD has been attempted to be ascertained and biopsy-proven stages of fibrosis were taken as the gold standard. By MRI data analysis and correlation of findings with histopathology, the present study attempts to validate MRI as a cost-effective non-invasive diagnostic imaging modality and attempt its possible replacement of liver biopsy in everyday clinical practice [6]. Therefore, it is of interest to describe Retrospective study on MRI accuracy in assessing liver fibrosis among patients with chronic liver disease.

## Materials and Methods:

This was a retrospective analysis in a tertiary care facility, from January 2018 to December 2023, reviewing the medical records of 150 CLD patients diagnosed with chronic liver disease. Only those patients who had both MRI and liver biopsy done within six months of each other were included. Ethical permission was obtained and patient confidentiality was ensured throughout the study. Inclusion criteria were all adult patients aged 18 years or more with a definitive diagnosis of CLD; who underwent MRI elastography and biopsy for fibrosis staging. There were some exclusion criteria that comprised patients whose medical records were missing, or they had contraindications to MRI, or they had acute hepatitis or hepatic malignancies. MRI data were acquired on a 1.5T or 3T scanner using methods such as MR elastography, T1/T2 mapping and diffusion-weighted imaging for the measurement of liver stiffness and tissue properties. The reference standard for histopathological findings from liver biopsies used the METAVIR scoring system to classify the stages of fibrosis as F0-F4. Sensitivity and specificity along with PPV, NPV analysis were conducted for MRI and significant fibrosis (≥ F2) cases. Spearman's correlation coefficient (r) was calculated in order to ascertain the relationship of MRI-based scores with histopathological stages while p < 0.05 was taken statistically significant.

## Results:

A total of 150 patients with chronic liver disease (CLD) were included in the study. The mean age of the patients was 52.3 ± 10.6 years, with a male predominance (65%). MRI elastography and histopathological findings showed a strong correlation for staging liver fibrosis, with significant accuracy in detecting higher fibrosis stages (≥F2). Table 1 (see PDF) summarizes the demographic and clinical characteristics of the study cohort, showing the distribution of CLD etiology and fibrosis stages. Table 2 (see PDF) compares MRI-based fibrosis stages with histopathological findings, showing high sensitivity and specificity for significant fibrosis (≥F2). Table 3 (see PDF) highlights the diagnostic accuracy of MRI elastography, demonstrating its reliability for assessing liver stiffness across fibrosis stages. Table 4 (see PDF) highlights the correlation between MRI elastography-derived liver stiffness measurements and histopathological fibrosis stages, showing a significant positive correlation (r = 0.87, p < 0.01). Table 5 (see PDF) compares the diagnostic performance of MRI elastography and diffusion-weighted imaging (DWI) in detecting significant fibrosis (≥F2), with MRI elastography demonstrating superior accuracy. Table 6 (see PDF) highlights the comparison of diagnostic performance across different MRI field strengths (1.5T vs. 3T), showing better results with 3T systems for staging liver fibrosis. Table 7 (see PDF) summarizes patient outcomes based on fibrosis stage, showing higher rates of hepatic complications (*e.g.*, ascites, variceal bleeding) in patients with advanced fibrosis (F3 and F4). Table 8 (see PDF) compares MRI elastography with transient elastography (TE) in terms of diagnostic accuracy for advanced fibrosis (≥F3), demonstrating the superior performance of MRI elastography. Table 9 (see PDF) illustrates the economic comparison between MRI and biopsy, showing MRI as a cost-effective alternative due to reduced procedural complications and shorter recovery times. Table 10 (see PDF) presents patient satisfaction rates, with higher satisfaction reported for MRI due to its non-invasive nature and absence of procedural risks.

The accuracy of MRI in the evaluation of liver fibrosis is summed up in ten tables in the study. Table 1 (see PDF) gives a summary of demographic and clinical characteristics; it was predominantly male and NAFLD was the leading cause of CLD. Table 2 (see PDF) and Table 3 (see PDF) indicate that the sensitivity of MRI elastography to diagnose significant fibrosis (≥F2) was 88% and the specificity was 91%. Table 4 (see PDF) presents a good correlation of MRI-derived liver stiffness with histopathologic fibrosis stages with an r = 0.87. Table 5 (see PDF) and Table 6 (see PDF) Compare the diagnostic performance of MRI elastography with DWI and shows that MRI elastography seems to perform better at 3T field strength. Table 7 (see PDF) shows higher the stage of fibrosis, the more it enhances the complications in the liver: 65% for F4. Table 8 (see PDF) Superior accuracy of MRI elastography compared to transient elastography (TE) in detecting advanced fibrosis Table 9 (see PDF) Economic aspects - MRI is cost-effective due to lower complications Table 10 (see PDF) Higher patient satisfaction with MRI because of its non-invasive nature and procedural safety. This summary highlights the reliability of MRI as a non-invasive, accurate and patient-preferred tool for the assessment of liver fibrosis, supporting its potential as an alternative to biopsy.

## Discussion:

This study of MRI of liver fibrosis in patients with chronic liver disease (CLD) demonstrates its high accuracy and reliability. Here, a sensitivity rate of 88% and specificity of 91% were achieved with MRI elastography as compared to histopathology, with excellent agreement as an accurate non-invasive alternative for liver biopsy [7]. The excellent correlation between the liver stiffness measurements derived using MRI with stages of fibrosis (r = 0.87) further emphasizes its potential as a diagnostic tool [8]. Advanced fibrosis (F3-F4) was associated with increased hepatic complications such as ascites and variceal bleeding, emphasizing the clinical importance of accurate staging [9]. MRI's superior performance over transient elastography (TE) and diffusion-weighted imaging (DWI) highlights its versatility in detecting significant and advanced fibrosis [10]. Additionally, MRI performed better at 3T field strength, indicating the advantages of higher resolution imaging in fibrosis assessment [11]. Economic analysis showed that MRI is cost-effective compared to biopsy since it has a lower rate of procedural complications and higher patient satisfaction. It is especially attractive to patients because 95% reported high satisfaction [12, 13]. Although MRI provides a safer and more efficient technique for the assessment of fibrosis, its implementation must consider accessibility and cost in resource-poor settings [14, 15]. Future studies should include increasing access to MRI and exploring the utility of MRI in monitoring progression of fibrosis and treatment response.

## Conclusion:

MRI and particularly MRI Elastography is a precise and reliable tool of diagnosing liver fibrosis in chronic liver disease (CLD). It is highly sensitive and specific with good correlation with histopathological findings. MRI offers a non-invasive alternative to replace liver biopsy with reduced procedure risks. It offers improved patient acceptability and enhanced cost-effectiveness. Its performance as a diagnostic improves with increased strengths of magnetic fields. MRI facilitates earlier diagnosis and more precise monitoring of the development of fibrosis. Its use can significantly extend clinical management in liver disease. There should be further studies aimed at improving access. Long-term validation in several different clinical settings is also required.
